# Position of Larval Tapeworms, *Polypocephalus* sp., in the Ganglia of Shrimp, *Litopenaeus setiferus*

**DOI:** 10.1093/icb/icu043

**Published:** 2014-05-11

**Authors:** Nadia Carreon, Zen Faulkes

**Affiliations:** *Department of Biology, The University of Texas-Pan American, 1201 W. University Drive, Edinburg, TX 78539, USA; ^†^Department of Biological Sciences, The University of Texas at Brownsville, One West University Boulevard - LHSB 2.816, Brownsville, TX 78520, USA

## Abstract

Parasites that invade the nervous system of their hosts have perhaps the best potential to manipulate their host’s behavior, but how they manipulate the host, if they do at all, could depend on their position within the host’s nervous system. We hypothesize that parasites that live in the nervous system of their host will be randomly distributed if they exert their influence through non-specific effects (i.e., general pathology), but that their position in the nervous system will be non-random if they exert their influence by targeting specific neural circuits. We recorded the position of larval tapeworms, *Polypocephalus* sp., in the abdominal ganglia of white shrimp, *Litopenaeus setiferus*. Tapeworms are more common within ganglia than in the section of the nerve cord between ganglia, even though the nerve cord has a greater volume than the ganglia. The tapeworms are also more abundant in the periphery of the ganglia. Because most synaptic connections are within the central region of the ganglion, such positioning may represent a trade-off between controlling the nervous system and damaging it.

## Introduction

Neurobiologists are impressed by the precision of neural connections. In invertebrates in particular, the number, type, and synaptic connections of neurons within a species are often highly specific. For example, the wild type of the nematode worm *Caenorhabditis elegans* has precisely 302 neurons, and the synaptic connections between all neurons are mapped (White et al. 1986). The chemical and electrical synapses of the stomatogastric ganglion of several crustacean species have been completely specified (Harris-Warrick et al. 1992; Katz and Tazaki 1992); that is, we have a connectome of that portion of the nervous system. The diameter of dendrites ranges from a few micrometers at the end near the cell body, to 10ths or 100ths of micrometers at the tip and dendritic spines (Fiala et al. 2008). The synaptic cleft, across which neuroactive chemicals diffuse, is about 20 nm wide (Ribrault et al. 2011). Even though there are very few species in which such a thorough description has been achieved, and accepting that the strength of those connections can be altered substantially (Kaas et al. 1983; Abbott and Nelson 2000; Fortin et al. 2012; Huganir and Nicoll 2013), there is no doubt that much of neural function at any given moment depends on the specified anatomical synaptic connections between neurons.

Given the specificity and small size of synaptic connections, it seems that having multicellular parasites living in a nervous system would be extremely likely to disrupt those connections. Previously, we found that larval tapeworms (*Polypocephalus* sp.) infect the central nervous system of white shrimp *Litopenaeus setiferus* (Carreon et al. 2011). *Polypocephalus* species do not always infect crustaceans (Cake 1979) or neural tissue (Brockerhoff and Jones 1995), but in this case, the degree of infection was correlated with the activity levels of the shrimp, with heavily infected shrimp walking more than less-infected ones (Carreon et al. 2011). These changes in behavior may make the shrimp more likely to be eaten by skates or rays, the probable definitive hosts of the tapeworm (Subhapradha and Hindle 1951; Caira et al. 1999; Call 2007; Koch 2009). Thus, this shrimp-tapeworm system is a potential case of parasite-induced trophic transmission (Lafferty 1999).

How tapeworms influence the shrimps’ behavior is not clear, but given that specific functions often are localized in particular regions of the nervous system, it is reasonable to hypothesize that the position of *Polypocephalus* sp. within the ganglia might be extremely specific, for two reasons. First, being closer to the functionally important area of the nervous system would allow a greater probability of manipulation (i.e., “access to the control panel”), particularly if the mechanism of manipulation involves secreting or altering neuroactive chemicals (Helluy and Holmes 1990; Adamo 2002; Helluy and Thomas 2003; Biron et al. 2005). Further, locations particularly advantageous for manipulation of the host might be preferred by parasites, and colonized first. Thus, individuals with low rates of infection might have parasites in more stereotyped locations that those with high numbers of parasites.

A second, competing consideration is that in the system of parasite-induced trophic transmission, the parasites must avoid key regions in the nervous system to prevent severe damage and killing the intermediate host before it can be eaten by the next host in the life cycle (usually the definitive host), so why are infected shrimp not completely debilitated by the presence of the parasites? The tapeworm larvae are ∼100 µm long (Carreon et al. 2011), which is about the same size as the largest cell bodies in the shrimps’ nervous system, and orders of magnitude larger than axons and dendrites. Parasites in neural tissue can damage it in many ways, including hemorrhaging and cellular degeneration (Sprent 1955). Non-random positioning of the tapeworms would be consistent with a “scalpel” tactic: tapeworms specifically exploit features of the host’s nervous system.

Alternately, it is possible that the tapeworms are located in the nervous system not because sites are targeted that facilitate manipulation of the host, but for other reasons, such as escaping from immune responses (Szidat 1969). In that case, the position of the tapeworms might be random within the nervous system. A random distribution between the ganglia and the cord connecting them, or a random distribution exclusively within the ganglia, would be consistent with a “shotgun” tactic, that is, tapeworms altering shrimps’ behavior through non-specific immune responses by the host (general pathology).

The general ground-plan of decapod crustacean nervous systems is highly conserved, allowing detailed comparisons to be made at the level of individual neurons (Arbas et al. 1991; Katz and Tazaki 1992). Like other arthropods (Bullock and Horridge 1965), *L. setiferus* has a ventral nerve cord consisting of a chain of ganglia. The cord is contained in a tough sheath, and how the tapeworm larvae penetrate the sheath is unknown. Each ganglion is associated with specific appendages (e.g., subesophageal ganglion with mouthparts, thoracic ganglion with walking legs, and abdominal ganglia with swimmerets), and contains sensory and motor neurons leading to each appendage and to the trunk of the body. The majority of synaptic connections are within the neuropils of each ganglion, which are roughly in the center of the ganglion, between a ventral rind of cell bodies and a dorsal set of axon-tracts that exit the ganglion (Skinner 1985a, 1985b; Kondoh and Hisada 1986; Leise et al. 1986, 1987; Mulloney et al. 2003). Each ganglion is separated by a cord that mostly contains axons, and few synaptic connections. In crayfish, an abdominal ganglion is estimated to contain about 600–700 neurons (Wine 1984; Kondoh and Hisada 1986); this number may be somewhat smaller in *L. setiferus*, based on comparisons of homologous pools of motor neurons (Faulkes 2007). Here, we examine the position of these parasites in the abdominal ganglia, the region of the nervous system where they are most abundant (Carreon et al. 2011).

## Materials and methods

Live white shrimp, *L. setiferus* (Linnaeus, 1767) were purchased from commercial suppliers in Port Isabel, TX, USA, and brought to the main campus of The University of Texas-Pan American in Edinburg, TX, USA. Shrimp were housed in a circulating seawater aquarium before being used.

To compare the number of tapeworms in the abdominal ganglia to the nerve cord, shrimp were anesthetized by chilling on ice. The anterior three abdominal ganglia were dissected, pinned in a dish lined with Sylgard (Dow Corning), then dehydrated in a progressive alcohol series (70%, 90%, and 100% ethanol for 5 min each, then 100% ethanol for 10 min). The dehydrated nerve cords were cleared in methyl salicylate and viewed on a compound light microscope.

We tested for differences in numbers of parasites in different regions of the nervous system with PASW Statistics 18 (SPSS, Inc.), using non-parametric statistics because the data were not normally distributed.

To determine the position of tapeworms in the abdominal ganglia, we examined the second abdominal ganglion, because the structure of the first five anterior abdominal ganglia is generally very similar (Mittenthal and Wine 1978; Wine 1984; Kondoh and Hisada 1986). The second abdominal ganglion was photographed at 10 µm intervals under a compound microscope. Only individuals in which parasites were seen within the ganglia were included in the analysis. The images from each individual were assembled into a composite image using Helicon Focus software (HeliconSoft Ltd). An outline of a ganglion was matched by eye to the composite, and lines were drawn to show each individual *Polypocephalus* sp. in the ganglion.

We estimated the relative proportions of the second and third abdominal ganglia, and the nerve cord connecting them, from photographs. The ganglia were estimated as spheres 750 µm in diameter, and the nerve cord was estimated as a cylinder 500 µm in diameter and 3 mm long.

## Results

**Fig. 1:**
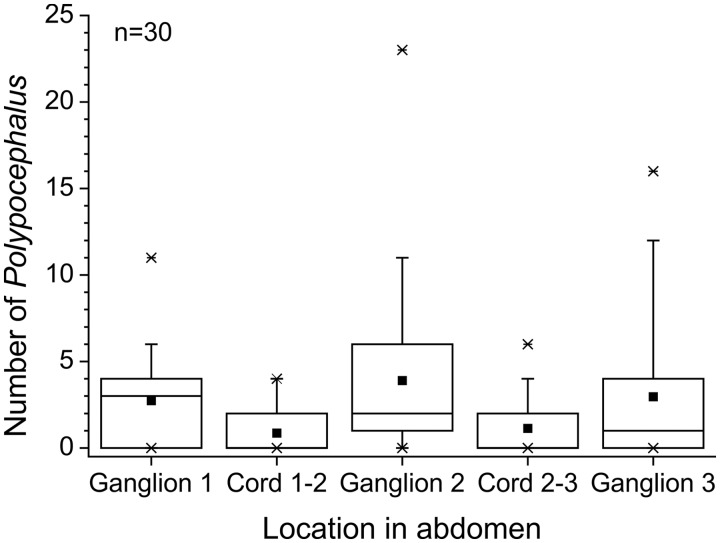
Number of *Polypocephalus* sp. individuals in the anterior portion of the abdominal nerve cord of *Litopenaeus setiferus*. Square = average; horizontal line = median; box = 50% of data; whiskers = 95% of data; crosses = minimum and maximum.

*Polypocephalus* sp. are found significantly more often in ganglia than in the nerve cord between ganglia (Fig. 1; Friedman test, *n* = 30, χ^2^ = 35.11, df = 4, *P* < 0.01), despite an abdominal ganglion having a smaller volume (estimated at 2.21 µl) than the nerve cord between them (estimated at 5.89 µl). Additionally, some larval tapeworms were seen in the nerves leading to the periphery, with the diameter of the nerve appearing about the same size, or even smaller, than the diameter of the larval tapeworm.

**Fig. 2:**
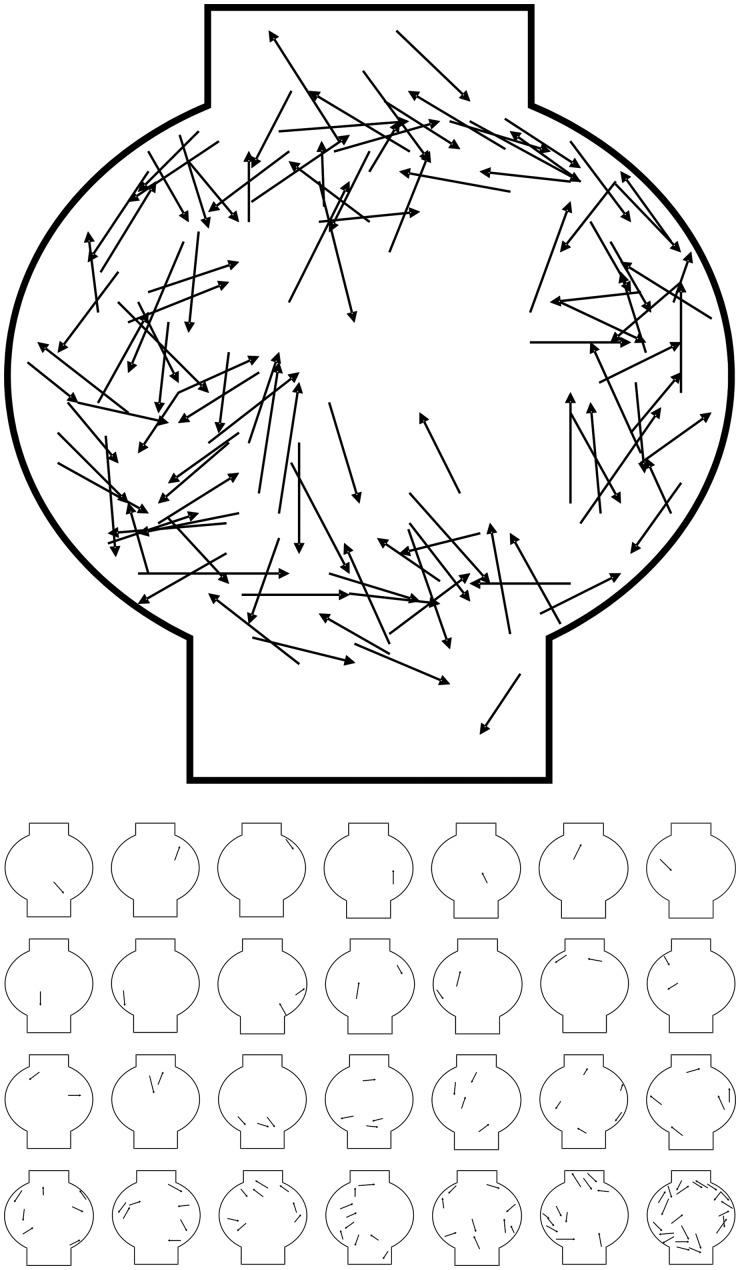
Positions of *Polypocephalus* sp. in a composite of abdominal ganglion 2 (*n *= 28 shrimp); arrowhead at anterior end of *Polypocephalus* sp. Thumbnails from each individual are shown below, arranged from fewest to most parasites.

*Polypocephalus* sp. are more often found in the margins of the ganglion (Fig. 2; *n* = 28 shrimp, 118 parasites). There is no location in the ganglion that is preferentially picked by single parasites (Fig. 2, thumbnails).

## Discussion

*Polypocephalus* sp. in the abdominal nerve cord of shrimp are found more often in ganglia than in the nerve cord, and more often in the periphery of the ganglia than in the center. This non-random positioning is consistent with the hypothesis that the tapeworms’ position in the nerve cord is related to their ability to manipulate the host. Sections of the abdominal ganglia are needed at higher resolution to more accurately determine the precise position of the parasites relative to the cell bodies, neuropil regions, and axon tracts, and to more closely assess whether there is any damage to the neural tissue.

That the position of the larval *Polypocephalus* sp. within a ganglion is not extremely specific is consistent with there being one or more trade-offs. It is potentially beneficial to the parasite to be within the ganglion rather than in the nerve cord between them, because there are few synaptic connections in the cord. This may benefit the tapeworms by increasing the probability of successfully manipulating the host, and increasing the influence exerted through that manipulation (i.e., effect size). Each individual tapeworm exerts only a small effect on the host (Carreon et al. 2011), like many manipulative parasites (Poulin 1994b). In general, host-manipulating parasites could benefit from infections by others of the same species, because each new individual increases the probability of the host engaging in the desired behavior, thereby increasing the chance of trophic transmission (Brown 1999; Shirakashi and Goater 2002; Brown et al. 2003). Being positioned deep near the center of the ganglion, however, may run the risk of disrupting synaptic connections, causing neuroinflammation (Helluy and Thomas 2010), and generally damaging neural tissue, as seen in other systems (e.g., Sprent 1955). Another possible cost might be a narrower range of potential hosts (Fredensborg 2014, this volume), although this cost may be small if the host has many closely related species and if the affected region of the nervous systems is generally similar, as is often the case (Kavanau 1990; Arbas et al. 1991). There may well be other-density-dependent costs (Poulin 1994a; Brown et al. 2003), but these may not be related to manipulation (Saldanha et al. 2009) and are probably incurred regardless of position in the nervous system.

An alternate hypothesis to explain the pattern of distribution within a ganglion is that the tapeworm larvae are found on the periphery of the ganglia because they tend to stop migrating deeper into the nervous system once they are past the sheath; that is, they are secure once inside the neural tissue. This would be consistent with the hypothesis that the nervous system is a place where immune responses are avoided (Szidat 1969). If the distribution of *Polypocephalus* sp. within the ganglion was not related to manipulation, however, yet another explanation would be required to explain why there are more parasites in the ganglion than in the cord.

Thus, *Polypocephalus* sp. larvae appear to use an intermediate infection-tactic, somewhere between a shotgun and a scalpel. Shotgun and scalpel strategies represent the two ends of a continuum; to continue the metaphor, an intermediate strategy might be termed a “club.” Although this host–parasite system involves many parasites, each with small effects, the same logic can be applied to single parasites that make increasingly large incursions into the body of their host via growth, such as fungal infections (*Ophiocordyceps* spp.) in ants (Hughes et al. 2011) or the interna of parasitic barnacles (*Sacculina* spp.) in crabs (Høeg 1995; Høeg and Lützen 1995; Shukalyuk 2002): a single parasite may be better able to manipulate its host by reaching deeper into the host’s body, but the extension increases the chance of killing the host before the parasite can complete the current stage of its life cycle. Thus, regardless of whether there is one parasite invading a host or many, the detailed spatial patterns of infection should provide clues to the mechanisms and strategies for manipulation.
